# Lignocellulose degradation at the holobiont level: teamwork in a keystone soil invertebrate

**DOI:** 10.1186/s40168-018-0536-y

**Published:** 2018-09-17

**Authors:** Marius Bredon, Jessica Dittmer, Cyril Noël, Bouziane Moumen, Didier Bouchon

**Affiliations:** 1 Laboratoire Ecologie et Biologie des Interactions-UMR CNRS 7267, Equipe Ecologie Evolution Symbiose-Batiment B8-B35, Université de Poitiers, 5 rue Albert Turpain, TSA 51106, F-86073 Poitiers Cedex 9, France; 2 Dipartimento di Biologia e Biotecnologie, Università degli Studi di Pavia, Pavia, Italy

**Keywords:** Microbiome, Transcriptome, CAZyme, Holobiont, Host–symbiont interactions, Isopods, RT-qPCR

## Abstract

**Background:**

Woodlice are recognized as keystone species in terrestrial ecosystems due to their role in the decomposition of organic matter. Thus, they contribute to lignocellulose degradation and nutrient cycling in the environment together with other macroarthropods. Lignocellulose is the main component of plants and is composed of cellulose, lignin and hemicellulose. Its digestion requires the action of multiple Carbohydrate-Active enZymes (called CAZymes), typically acting together as a cocktail with complementary, synergistic activities and modes of action. Some invertebrates express a few endogenous lignocellulose-degrading enzymes but in most species, an efficient degradation and digestion of lignocellulose can only be achieved through mutualistic associations with endosymbionts. Similar to termites, it has been suspected that several bacterial symbionts may be involved in lignocellulose degradation in terrestrial isopods, by completing the CAZyme repertoire of their hosts.

**Results:**

To test this hypothesis, host transcriptomic and microbiome shotgun metagenomic datasets were obtained and investigated from the pill bug *Armadillidium vulgare*. Many genes of bacterial and archaeal origin coding for CAZymes were identified in the metagenomes of several host tissues and the gut content of specimens from both laboratory lineages and a natural population of *A. vulgare*. Some of them may be involved in the degradation of cellulose, hemicellulose, and lignin. Reconstructing a lignocellulose-degrading microbial community based on the prokaryotic taxa contributing relevant CAZymes revealed two taxonomically distinct but functionally redundant microbial communities depending on host origin. In parallel, endogenous CAZymes were identified from the transcriptome of the host and their expression in digestive tissues was demonstrated by RT-qPCR, demonstrating a complementary enzyme repertoire for lignocellulose degradation from both the host and the microbiome in *A. vulgare*.

**Conclusions:**

Our results provide new insights into the role of the microbiome in the evolution of terrestrial isopods and their adaptive radiation in terrestrial habitats.

**Electronic supplementary material:**

The online version of this article (10.1186/s40168-018-0536-y) contains supplementary material, which is available to authorized users.

## Background

Plant biomass decomposition represents a key step in the terrestrial carbon cycle [1, 2] that is carried out by the combined action of fungi, microbes, and decomposer animals such as “litter transformer” macroarthropods [3, 4]. Plants, and by extension dead plant biomass, are mostly composed of lignocellulose, making it the most abundant biomass component on Earth. Thus, the process of lignocellulose degradation is of great research interest, especially for biotechnology, due to its potential as a sustainable resource for biofuels and biomaterial production [5–7].

Lignocellulose is composed of cellulose, hemicellulose, and lignin. The degradation of these polymers requires the synergistic action of multiple Carbohydrate-Active enZyme (called CAZymes) families, typically acting together as an enzyme cocktail with multiple complementary and coordinated oxidative, hydrolytic and non-hydrolytic activities [7, 8]. Since lignin, a complex heteropolymer providing strength and resistance to plant tissues [9], protects carbohydrate polymers against enzymatic digestion, its degradation is a critical first step in lignocellulose degradation enabling the liberation of cellulose and hemicellulose [10, 11]. Many enzymes are known as lignin-modifying enzymes (LMEs): lignin peroxidases, manganese peroxidases, versatile peroxidases, laccases and cellobiose dehydrogenases [12]. Cellulose, on the other hand, is a polymer of several glucose monomers linked by ß-1,4-glycosidic bonds, necessary for the rigid structure of plant cell walls [13]. Commonly, a set of three enzymes is required for the hydrolysis of cellulose into glucose monomers: (1) endoglucanases, (2) exoglucanases, and (3) β-glucosidases [14]. Finally, hemicellulose is a complex polysaccharide with large variations both within and between plant species and plant tissues. Hemicellulases can be classified in three types [15]: (1) endo-hemicellulases which cleave the main chain internally, (2) exo-hemicellulases which release monomeric sugars, and (3) debranching enzymes (also known as accessory enzymes) which cleave the side chains of the polymers or associated oligosaccharides.

In nature, fungi and bacteria are the main producers of enzymes which decompose lignocellulose, making them the most important players in plant biomass degradation [16, 17]. Lignocellulose decomposition is a rare trait among animals, since most plants have evolved structural and chemical mechanisms of resistance against attacks by herbivores [6]. Furthermore, there is no animal genome known to date that encodes all necessary enzymes to break down plant polysaccharides into sugar monomers [18]. Many animals possess a few lignocellulose-degrading enzymes [19, 20], but in most cases, an efficient degradation of lignocellulose is only achieved through mutualistic associations with microbial symbionts [21]. Lignocellulose degradation in these animals is thus achieved at the holobiont level (i.e., a host and its associated microbiota [22]), relying on the complementary action of the lignocellulose-degrading enzyme repertoire from the host and its associated microbial symbionts. In invertebrates, termites represent the most studied model system for the process of lignocellulose degradation. Due to a spatial, complementary and synergistic cooperation between the host and its microbiome [23–26], termites are able to digest lignocellulose with a high efficiency, making them one of the most powerful animal species for this process [27, 28]. Other macroarthropods such as millipedes [29] or terrestrial isopods [30, 31] are also known to contribute significantly to the decomposition of lignocellulose, but the respective roles of the host and the symbionts for lignocellulose digestion in these species are still unknown.

Terrestrial isopods are recognized as keystone species in terrestrial ecosystems due to their important role in the decomposition of organic matter [32]. They contribute directly to litter decomposition and nutrient cycling by digesting substrates [33–35], and indirectly through their feces which affect the soil microbial community and its activity [30, 36, 37]. Zimmer et al. [38, 39] hypothesized that the gut microbiota had facilitated or even enabled the colonization of land by terrestrial isopods, by contributing the necessary enzymes for the digestion of terrestrial food sources. Specifically, it has been suggested that terrestrial isopods are able to exploit lignocellulose with the help of hepatopancreatic (i.e., resident in the caeca) and/or environmental bacteria [38–42]. While several bacterial symbionts were indeed identified in the caeca of diverse isopod species (i.e., *Candidatus* Hepatincola porcellionum [43] and *Candidatus* Hepatoplasma crinochetorum [44, 45]) and were initially hypothesized to be involved in lignocellulose digestion (reviewed in [42, 46]), their exact functions within the isopod holobiont still remain to be elucidated. Moreover, the subsequent discovery of endogenous cellulases in isopods [47–49] raised questions regarding the role of the isopod gut microbiota in lignocellulose digestion.

In the present study, we address this question by combining for the first time transcriptomic and shotgun metagenomic approaches in the common pill-bug *Armadillidium vulgare.* This species is known to harbor a high diversity of bacteria in various tissues [50–52] and thus represents an excellent model to study diverse symbiotic interactions [42]. To this end, metagenomes from several tissues and the gut content of specimens from both laboratory lineages and a natural population were sequenced and used to identify lignocellulose-degrading CAZymes of prokaryotic origins. In parallel, host lignocellulose-degrading CAZymes were identified in the transcriptome of *A. vulgare*, and their expression in host tissues was verified by RT-qPCR. This work allowed us to (i) describe the enzyme repertoire implicated in lignocellulose degradation in the *A. vulgare* holobiont, (ii) identify microbial taxa contributing lignocellulose-degrading genes, and (iii) unveil potential interactions between host and symbionts enabling an efficient lignocellulose digestion.

## Methods

### Biological samples

Transcriptomic data were generated from *A. vulgare* females originating from 10 populations that are maintained in the laboratory in population cages (Table 1). In the laboratory, all animals were kept at 20 °C and natural photoperiod in plastic breeding boxes on moistened potting mix and fed ad libitum with lime tree leaves and carrot slices.

**Table 1:** *A. vulgare* samples used for the reference transcriptome and assembly statistics

Samples					
Origin	*Wolbachia* status	Gender	*N*	GenBank	No. of reads
Celles sur Belle, France	+	F	1	SRS625835	3,881,922
Vancouver, Canada	+	F	1	SRS625837	7,081,881
Mentrida, Spain	–	F	1	SRS625838	8,768,792
Heraklion, Greece	–	F	1	SRS625839	28,956,650
Fornazo, Italia	–	F	1	SRS625840	8,826,638
Helsingör, Denmark	+	F	1	SRS625841	7,520,054
Porto Alegre, Brazil	–	F	1	SRS625842	13,957,815
Germany	–	F	1	SRS625843	8,824,057
Prague, Czech Republic	+	F	1	SRS625844	35,468,027
Saint Guilhem, France	+	F	1	SRS625845	10,874,926
Total					134,160,762
Statistics					
Parameters				Number of sequences	
Total number of bases				104,530,589	
Total number of transcripts				143,383	
Total number of transcripts after removing Prokaryotes and viruses				142,909	
Mean GC count				36%	
N50				1805	
Mean length				685	
% reads used for assembly				95.85%	
Total number of predicted ORFs				43,672	

Metagenomic data were generated from two laboratory lineages (one *Wolbachia*-free, the other harboring the feminizing strain *w*VulC, *N* = 22 individuals) and one field population (10 males and 10 females) of *A. vulgare* (Table 2). Individuals from the field were collected in 2012 and 2014 at Availles (France, 46° 51′ 37′′ N, 0° 8′ 28″ E) and were kept in plastic boxes with soil and leaves from their respective sampling site until dissection within 2 days after collection. Isopods collected in 2012 were the same as in our previous metabarcoding studies [51, 52]. *Wolbachia* infection status was determined for all individuals via PCR and sequencing of the *wsp* gene.

**Table 2:** Metrics of the *A. vulgare* metagenome assemblies

Origin	*N*	Gender	*Wolbachia*	Tissues	No. of reads	No. of contigs	N50	% reads used	No. of predicted ORFs^1^	No. of LDC*
Laboratory	7	M	–	Tissue samples*	93,016,946	545,551	575	79.3	173,550	1
Laboratory	7	M	–	Hindgut	151,479,496	862,485	745	85.2	300,044	1
Laboratory	7	M	–	Gut content	44,025,120	181,858	490	60.5	77,326	135
Laboratory	8	F	–	Tissue samples*	115,690,244	617,032	638	82.6	198,980	0
Laboratory	8	F	–	Hindgut	213,961,342	919,075	727	86.1	309,351	4
Laboratory	8	F	–	Gut content	85,069,086	460,039	516	70	165,226	92
Laboratory	7	F	+	Tissue samples*	136,360,100	714,448	647	84.5	238,023	32
Laboratory	7	F	+	Hindgut	196,487,170	960,311	760	90	321,849	6
Laboratory	7	F	+	Gut content	61,471,292	284,429	502	65.1	101,524	72
Availles	10	M	–	Tissue samples*	133,995,390	708,519	707	85.7	229,060	2
Availles	10	M	–	Hindgut	211,001,880	857,462	747	87.7	286,800	7
Availles	10	M	–	Gut content	36,755,870	19,887	513	10.1	20,111	46
Availles	10	F	+	Tissue samples*	144,043,638	692,481	658	85.7	230,975	13
Availles	10	F	+	Hindgut	212,185,430	920,773	814	90.4	314,895	17
Availles	10	F	+	Gut content	55,605,558	200,488	452	52.7	67,468	36

*LDC*, lignocellulose-degrading CAZymes

*Tissue samples: caeca, nerve cords, gonads, and hemolymph

^1^ORFs, predicted ORFs after filtering

### Transcriptomics: RNA extraction and sequencing

Total RNA was extracted from one whole adult female from each population. Each individual was frozen in liquid nitrogen and grinded with a mortar and pestle. The resulting powders were processed using TRIzol® Reagent (Invitrogen) to extract RNA according to the manufacturer’s protocol. Quantity and quality of total RNA were determined using agarose gel electrophoresis, NanoDrop spectrophotometer (ThermoFisher) and Bioanalyzer (Agilent). The extracted materials were stored at − 80 °C until use.

Single-end sequencing libraries were constructed, after mRNA enrichment based on the existence of poly(A) tails and sequenced by the “Institut des Sciences et de l’Évolution” (Montpellier, France) on the Illumina HiSeq 2000 sequencing platform to produce 50 bp single-end reads. These reads have been used previously in a transcriptome-based population genomics study [53]. The total number of reads obtained ranged from 3,881,922 to 35,468,027 depending on the library (Table 1). The raw reads are available in SRA archive under accession numbers SRS625835 and SRS625837-SRS625845.

### De novo host transcriptome assembly

Read quality was checked with FastQC (version 0.11.2; http://www.bioinformatics.babraham.ac.uk/projects/fastqc). To identify and remove mitochondrial contaminants, reads were aligned against the *A. vulgare* mitogenome (accession number EF643519.3) using Bowtie (version 1.1.0; [54]). Removal of sequencing adaptors and undetermined nucleotides were performed with FASTX-Toolkit (version 0.0.13; http://hannonlab.cshl.edu/fastx_toolkit/index.html) and Cutadapt (version 1.9; [55]). Reads shorter than 35 bp were removed. Cleaned reads from each library were assembled using Velvet (version 1.2.08; [56]) and Oases (version 0.2.08; [57]) with 11 values of *k*-mers (27–47). Assemblies were then merged, and redundancy was removed by clustering transcripts with ≥ 90% identity from each *k*-mer using CD-HIT-EST (version 4.6; [58]).

To remove transcripts corresponding to possible prokaryotes and viruses, transcripts were compared with the non-redundant protein database (1 March 2017) using BLASTx [59] with an *E* value cut-off of 0.0001. All transcripts identified as prokaryotes or viruses were removed from the transcriptome. The quality of the resulting assembly was assessed with BUSCO (version 3.0.1; [60]) referring to core arthropod genes. The coverage of each transcript was calculated as reads per kilo base per million mapped reads (RPKM [61]).

### Metagenomics: DNA extraction and sequencing

Prior to dissection, all individuals were surface sterilized using sodium hypochlorite and hemolymph was collected after piercing the cuticle with a sterile needle. Several tissues (i.e., hindgut, caeca, nerve cords, and gonads) were then dissected out using sterilized instruments and rinsed in Ringer solution to avoid cross-contamination between tissues. In order to separate the hindgut tissue from the gut content, the bulk of the gut content was squeezed out of the hindgut into a 1.5 ml microcentrifuge tube containing extraction buffer (100 mM Tris [pH 8.0], 100 mM EDTA, 100 mM NaCl, 0.1% SDS, 50 mM DTT, 1.25% Proteinase K) using sterile forceps. Subsequently, the hindgut was cut longitudinally and washed in Ringer solution to remove remaining gut content.

All hemolymph, tissue, and gut content samples were then homogenized in extraction buffer, and total DNA was purified using phenol-chloroform [62]. Equimolar amounts of DNA from 7 to 10 biological replicates of the same tissue and sample type (i.e., origin, gender, *Wolbachia* infection status) were pooled and prokaryotic DNA was enriched in each pool using the NEBNext® Microbiome DNA Enrichment kit (New England Biolabs) according to the manufacturer’s instructions. The enriched DNA was quantified using PicoGreen (Invitrogen). To reduce the number of samples for sequencing, enriched DNA from hemolymph, gonads, nerve cords, and caeca (i.e., all tissues except the hindgut) were pooled in equimolar amounts for each sample type (hereafter referred to as “tissue samples”), while enriched DNA from the hindgut and the gut content were kept as separate samples. This resulted in 15 shotgun metagenomic libraries which were sequenced on an Illumina HiSeq 2500 by GenoScreen (Lille, France), generating 2 × 100 bp paired-end reads. The total number of reads obtained for each library ranged from 36,755,870 to 213,961,342 (Table 2).

### Metagenomic shotgun data analysis

Read quality was checked with FastQC (version 0.11.2) and low-quality reads and sequencing adaptors were removed using Trimmomatic (version 0.32; [63]). Trimmed reads shorter than 35 bp were discarded. High quality reads from each library were assembled using MegaHit (version 1.0.3; [64]) with default parameters.

To remove host, eukaryote and virus contigs from the metagenome assemblies, contigs were searched against the *A. vulgare* reference transcriptome and the non-redundant protein database (1 April 2017) using BLASTn and BLASTx, respectively [59]. The minimum *E* value was set at 0.0001, and all contigs that matched to viruses, *A. vulgare* and other eukaryotes, were removed from the final metagenome assemblies.

### Carbohydrate-Active enZyme annotation

*A. vulgare* transcripts and metagenomic contigs encoding CAZymes were identified using the Carbohydrate Active enZymes (CAZy) database [65]. Prior to identification, all open reading frames (ORFs) were predicted for both the *A. vulgare* reference transcriptome and the metagenome assemblies using Transdecoder (version 3.0.1; https://github.com/TransDecoder/) and MetaProdigal (version 2.60; [66]), respectively, with default parameters for both. Subsequently, dbCAN [67], a database which uses hidden Markov models to define the signature domains for every CAZy family (i.e., glycoside hydrolases (GHs), Glycosyltransferases (GTs), polysaccharide lyases (PLs), carbohydrate esterases (CEs), auxiliary activities (AAs), and carbohydrate-binding modules (CBMs)) was used to identify CAZymes. All predicted ORFs were analyzed with dbCAN (1 March 2017) using HMMER (version 3.1b2; [68]) with an *E* value threshold of 0.0001. ORFs identified as CAZymes were then imported into Hotpep [69] to predict their enzymatic activity.

### Taxonomic assignment of identified CAZymes

For the genes annotated as CAZymes in metagenome assemblies, searches against the non-redundant protein database (1 April 2017) were performed using BLASTp [59]. An *E* value cut-off of 0.0001 was used and the top five hits were kept. The BLAST outputs were then imported into MEGAN6 software [70] for taxonomic assignment using the NCBI taxonomy database. Each ORF was thus assigned to the most accurate taxonomic rank (i.e., kingdom, phylum, class, order, family, genus, and species) based on the LCA (i.e., lowest common ancestor) algorithm. Results were visualized using the Phyloseq R package [71] and Circos software [72].

### Quantitative RT-PCR

The expression of 16 transcripts representing all lignocellulose-degrading CAZy families identified in the host transcriptome was verified by real-time quantitative reverse transcription PCR (RT-qPCR). Annotated transcripts with high RPKM values were chosen as representative of a given family. Three males and three females from Celles sur Belle, Heraklion, and Prague (Table 1) populations were used for dissections. Total RNA was extracted from their digestive tissues (i.e., caeca, hindgut, hindgut content) as well as non-digestive tissues (i.e., gonads, nerve cords, fat tissues) as described above for transcriptome sequencing. First-strand cDNA was synthesized using the SuperScript™ IV First-Strand Synthesis System kit (Invitrogen) according to the manufacturer’s protocol, with 500 ng of total RNA as template and using random hexamer primers. Specific primers for genes of interest were designed based on our transcripts with PRIMER3 software [73]. Gene-specific primers are listed in Additional file 1: Table S1. Quantitative RT-PCR was performed using the LightCycler LC480 system (Roche) as follows: 10 min at 95 °C followed by 45 cycles of 10 s at 95 °C, 10 s at 60 °C, and 20 s at 72 °C. A melting curve (65 °C to 97 °C) was recorded at the end of each reaction to check that the PCR product was unique. Each reaction mixture contained 6 μl SYBR Green I Master Mix (Roche), 0.6 μl of each forward and reverse primer (10 μM), 2.4 μl of nuclease-free water and 1.5 μl of cDNA template. Expression levels of target genes were normalized based on the expression level of two reference genes: Ribosomal Protein L8 (RbL8) and Elongation Factor 2 (EF2) [74]. Gene expression levels in the different tissues were compared using the nonparametric Kruskal–Wallis rank sum test in combination with Dunn’s post hoc multiple comparison test with Benjamini-Hochberg correction (PMCMR R package, R software version 3.4.0; https://www.r-project.org/).

## Results

### Quality of transcriptome and metagenome assemblies

The reference transcriptome of *A. vulgare* was produced using Illumina short reads technology in single-end mode, from 10 libraries generating a total of 134,160,762 reads (Table 1). After assembly, 143,383 transcripts were obtained with an N50 of 1805. Identified transcripts from prokaryotes and viruses (441 and 33 respectively) were removed from the assembly. Statistics of the final transcriptome are given in Table 1. Assembly completeness was evaluated using the BUSCO pipeline [60]. From the 1066 single-copy orthologous arthropod genes contained in the BUSCO database, 1021 (95.7%) complete genes (638 single-copy genes and 383 duplicated) and 25 fragmented genes (2.3%) were represented in the *A. vulgare* reference transcriptome assembly and only 20 genes were missing (2%). Thus, the multiple *k*-mer method we used for the assembly of the *A. vulgare* reference transcriptome led to a highly complete transcriptome.

The 15 metagenomes obtained from different host tissues as well as the gut content of *A. vulgare* generated a total of 1,891,148,562 Illumina reads. These were assembled into 19,887–960,311 contigs depending on the library (Table 2). The gut content metagenomes had lower numbers of reads, assembled contigs, and N50 values compared to metagenomes from host tissues (Table 2), indicating their complexity and taxonomic richness. In contrast, there was no difference in the number of reads, assembled contigs and N50 for metagenomes obtained from the hindgut and tissue samples. Furthermore, there was no difference between laboratory and field metagenome assemblies.

### Identification of CAZymes in the *A. vulgare* holobiont

The *A. vulgare* reference transcriptome assembly and the metagenome assemblies were screened for genes encoding CAZymes (Carbohydrate Active enZymes). Since CAZymes are characteristically modular in structure, and each CAZyme can contain several modules with distinct functions, we refer to modules rather than to the proteins in which they are contained. CAZy modules typically retain their functions when they are expressed, independent of the remaining protein regions. The dbCAN pipeline identified 1933 CAZy modules in the *A. vulgare* reference transcriptome and 3421 CAZy modules in the 15 metagenome assemblies (Additional file 2: Table S2; Additional files 3 and 4). RPKM values for CAZymes identified in the host transcriptome are given in Additional file 5: Table S3. The CAZyme-associated genes were classified into enzyme families according to the CAZy nomenclature (http://www.cazy.org/; [65]). A total of 231 CAZy families were identified in the *A. vulgare* holobiont, distributed across all known CAZy classes (i.e., GHs, GTs, PLs, CEs, AAs, and CBMs) (Additional file 2: Table S2). Among them, 36 and 133 families were specific to the host and its microbiome, respectively, and 62 families were present in both. Enzymatic activities of the identified CAZymes were predicted by Hotpep and listed in Additional file 6: Table S4.

The carbohydrate-binding modules (CBMs) were the most prevalent class in the *A. vulgare* holobiont with 63 different families, corresponding to 1200 and 1017 modules in the microbiome and the host, respectively. Among the most abundant CAZymes, the CBM14 family, a chitin binding module, was prominent in the host with 940 modules representing 48.6% of the total host CAZy modules, while it was only represented by 26 modules in the microbiome (0.008% of the microbiome CAZy modules). In contrast, the CBM47 family, a fucose-binding module, represented 18.4% of the total microbiome CAZy modules with 629 modules, whereas it represented only 0.004% of CAZy modules with 8 modules in the host transcriptome. The second most prominent class of CAZymes in the *A. vulgare* holobiont was the class of Glycosyltransferases (GTs), with 866 and 338 modules in the microbiome and the host, respectively, together representing 63 different families. Eight hundred eighty-seven modules in the microbiome and 403 in the host were associated with the class of glycoside hydrolases (GHs), distributed across 72 families. GHs with known chitinase and lysozyme activities were the most abundant both in the microbiome (GH23 = 119 modules) and in the host (GH18 = 128 modules). Moreover, 13 families of carbohydrate esterases (CEs) were represented by 328 and 155 modules in the microbiome and in the host, respectively. Among these, CE1 and CE10 families were the most abundant CE families in the *A. vulgare* holobiont, together representing 76.8% (119 modules) of the CEs in the host and 45.4% (149 modules) in the microbiome. The class auxiliary activities (AAs) was represented by 6 families, accounting for 85 modules in the microbiome and 17 modules in the host. Finally, polysaccharide lyases (PLs) belonging to 14 different PL families were the least abundant, with 55 modules in the microbiome and only 3 in the host.

### Lignocellulose-degrading CAZymes

Selected CAZymes known as lignocellulose-degrading enzymes and CBMs known as lignocellulose-binding modules were then examined in depth, due to their potential nutritional role in *A. vulgare*. In total, we identified 707 modules corresponding to a total of 38 lignocellulose-degrading CAZy families in the *A. vulgare* holobiont (506 modules in the microbiome and 201 in the host, Table 3). Among these, 21 families were specific to the microbiome, 4 were found only in the host, and 13 were present in both. A comparison of the glycoside hydrolase (GH) profiles of the *A. vulgare* microbiome and other animal gut microbiomes (inspired by the classification of Cardoso et al. [75] and Allgaier et al. [76]) is given in Additional file 7: Table S5. Overall, the GH profile of the *A. vulgare* microbiome is very similar to other animal gut microbiomes, except for mannases and debranching enzymes: the former are more abundant in the *A. vulgare* microbiome compared to other organisms, while the latter are less abundant. In addition, we identified 932 modules corresponding to 39 lignocellulose-binding module families (875 modules in the microbiome and 57 in the host, Table 3).

**Table 3:** List of CAZymes implicated in lignocellulose degradation in the *A. vulgare* holobiont. Presented are the total numbers of CAZy modules for each family in the host reference transcriptome and in the metagenome assemblies

CAZy family	Known activities	Host	Metagenome assemblies		
			Field	Lab	Total
LMEs		15	4	25	29
AA1	Laccase	2	–	–	*–*
AA2	Manganese peroxidase; versatile peroxidase; lignin peroxidase	–	–	16	16
AA3	Cellobiose dehydrogenase	13	4	9	13
Hemicellulases		121	86	226	321
CE1	Acetyl xylan esterase; feruloyl esterase	33	29	61	90
CE3	Acetyl xylan esterase	19	7	19	26
CE4	Acetyl xylan esterase	13	23	32	55
CE5	Acetyl xylan esterase	–	1	1	2
CE6	Acetyl xylan esterase	–	–	2	2
CE7	Acetyl xylan esterase	–	4	1	5
CE12	Acetyl xylan esterase	2	1	5	6
GH2	β-galactosidase; β-mannosidase; α-L-arabinofuranosidase	14	–	12	12
GH4	α-galactosidase	–	1	38	39
GH10	Endo-1,4-β-xylanase	–	1	–	1
GH11	Endo-β-1,4-xylanase	–	–	1	1
GH16	Xyloglucanase	1	2	2	4
GH27	α-galactosidase	5	–	–	*–*
GH29	α-L-fucosidase	18	2	–	2
GH31	α-galactosidase; α-xylosidase	–	6	23	29
GH35	β-galactosidase	12	–	–	*–*
GH36	α-galactosidase	–	3	2	5
GH39	β-xylosidase	–	–	3	3
GH42	β-galactosidase	–	7	2	9
GH43	β-xylosidase; α-L-arabinofuranosidase; arabinanase; xylanase	–	2	15	17
GH53	Endo-β-1,4-galactanase	–	–	2	2
GH57	α-galactosidase	–	2	–	2
GH113	β-mannanase	–	2	3	5
GH116	β-xylosidase	2	–	–	*–*
GH120	β-xylosidase	2	–	1	1
GH134	Endo-β-1,4-mannanase	–	–	3	3
Hemicellulases and/or cellulases		65	42	114	156
GH1	β-glucosidase; β-galactosidase; exo-β-1,4-glucanase; β-mannosidase; β-xylosidase	–	4	53	57
GH3	β-glucosidase; exo-β-1,4-glucanase; xylan 1,4-β-xylosidase; α-L-arabinofuranosidase	–	15	28	43
GH5	Endo-β-1,4-glucanase; β-glucosidase; exo-β-1,4-glucanase; endo-β-1,4-xylanase; β-mannosidase; endo-β-1,4-mannosidase; cellobiohydrolase	11	14	5	19
GH8	Endo-β-1,4-glucanase; endo-1,4-β-xylanase	–	–	21	21
GH9	Endo-β-1,4-glucanase; β-glucosidase; exo-β-1,4-glucanase; cellobiohydrolase	14	–	2	2
GH30	β-glucosidase; endo-β-1,4-xylanase; β-xylosidase	39	6	–	6
GH51	Endo-β-1,4-glucanase; endo-β-1,4-xylanase; β-glucosidase; β-xylosidase; α-L-arabinofuranosidase	–	1	–	1
GH74	Endo-β-1,4-glucanase; xyloglucanase	1	2	2	4
GH94	Cellobiose phosphorylase	–	–	3	3
Lignocellulose-binding modules		57	519	356	875
CBM1	Cellulose-binding	–	–	2	2
CBM2	Cellulose and xylan binding	–	4	2	6
CBM3	Cellulose-binding	–	3	1	4
CBM4	Cellulose, xylan, β-1,3-glucan, and β-1,3-1,4-glucan binding	–	1	1	2
CBM6	Cellulose-binding	–	3	1	4
CBM8	Cellulose-binding	–	–	1	1
CBM10	Cellulose-binding	–	1	1	2
CBM13	Xylan-binding	16	3	1	4
CBM15	Xylan and xylooligosaccharides binding	–	2	–	2
CBM16	Cellulose and glucomannan binding	–	–	2	2
CBM22	Xylan-binding	–	2	2	4
CBM23	Mannan-binding	1	1	–	1
CBM27	Mannan-binding	–	1	2	3
CBM29	Mannan and glucomannan binding	–	2	5	7
CBM30	Cellulose-binding	–	6	5	11
CBM31	β-1,3-xylan-binding	1	4	5	9
CBM32	Galactose-binding	19	9	42	51
CBM35	Xylan, mannans and β-galactan binding	–	4	2	6
CBM36	Xylans and xylooligosaccharides binding	–	4	2	6
CBM37	Cellulose and xylan binding	8	3	8	11
CBM39	β-1,3-glucan-binding	–	4	3	7
CBM42	Arabinofuranose-binding	–	1	1	2
CBM43	β-1,3-glucan-binding	–	4	1	5
CBM44	Cellulose and xyloglucan binding	–	8	8	16
CBM46	Cellulose-binding	–	2	4	6
CBM47	Fucose-binding	8	401	228	629
CBM49	Crystalline cellulose binding	–	5	4	9
CBM51	Galactose-binding	–	4	1	5
CBM54	Xylan-binding	–	6	1	7
CBM56	β-1,3-glucan-binding	1	–	1	1
CBM62	Xyloglucan, arabinogalactan, and galactomannan binding	–	1	–	1
CBM63	Cellulose-binding	–	2	2	4
CBM64	Cellulose-binding	–	5	2	7
CBM67	L-rhamnose-binding	3	4	6	10
CBM72	Cellulose, β-1,3-glucans, xylan, and β-mannan binding	–	5	2	7
CBM76	Xyloglucan, glucomannan, and β-glucan binding	–	1	1	2
CBM78	β-1,4-glucans and xyloglucan binding	–	1	2	3
CBM79	β-glucans-binding	–	3	–	3
CBM80	Xyloglucan, glucomannan, and galactomannan binding	–	9	4	13

The majority of the lignocellulose-degrading CAZymes identified in the microbiome were found in the gut content, representing 82.2% (*N* = 416) of the modules identified as cellulases, hemicellusases, and lignin-modifying enzymes (LMEs), whereas only 7.1% of the modules were identified in the hindgut (*N* = 36) and 10.7% in the tissues (*N* = 54) (Table 4). Among the 34 lignocellulose-degrading CAZy families found in the microbiome, 17 were present in all samples (tissue samples, hindgut, and gut content) and 14 were specific to the gut content. Three families were not found in the gut content: GH11 was specific to the hindgut, GH29 was specific to the tissue samples, and GH30 was found in the hindgut and the tissue samples. Concerning the lignocellulose-binding modules found in the microbiome, 40.1% (351 modules) were identified in the hindgut, 36.5% (319 modules) were identified in the tissue samples, and 23.4% (205 modules) in the gut content (Table 4). Twenty-one different lignocellulose-binding module families were found in the gut content and 5 of these were not detected in the microbiota from host tissues (CBM13, CBM16, CBM22, CBM51, CBM56). Twenty-seven families were found in the tissue samples (3 of which exclusively in this sample type: CBM1, CBM62, CBM8), and 29 families were found in the hindgut, again 3 of them being specific to this tissue (CBM10, CBM23, CBM42).

**Table 4:** Tissue distribution of CAZymes implicated in lignocellulose degradation in the metagenomes. Presented are the total numbers of CAZy modules for each family per sample type

CAZymes	Gut content	Hindgut	Tissue samples
Lignin-modifying enzymes	28	0	1
Hemicellulases	252	35	34
Hemicellulases and/or cellulases	129	8	19
Lignocellulose-binding modules	205	351	319

**Fig. 1:**
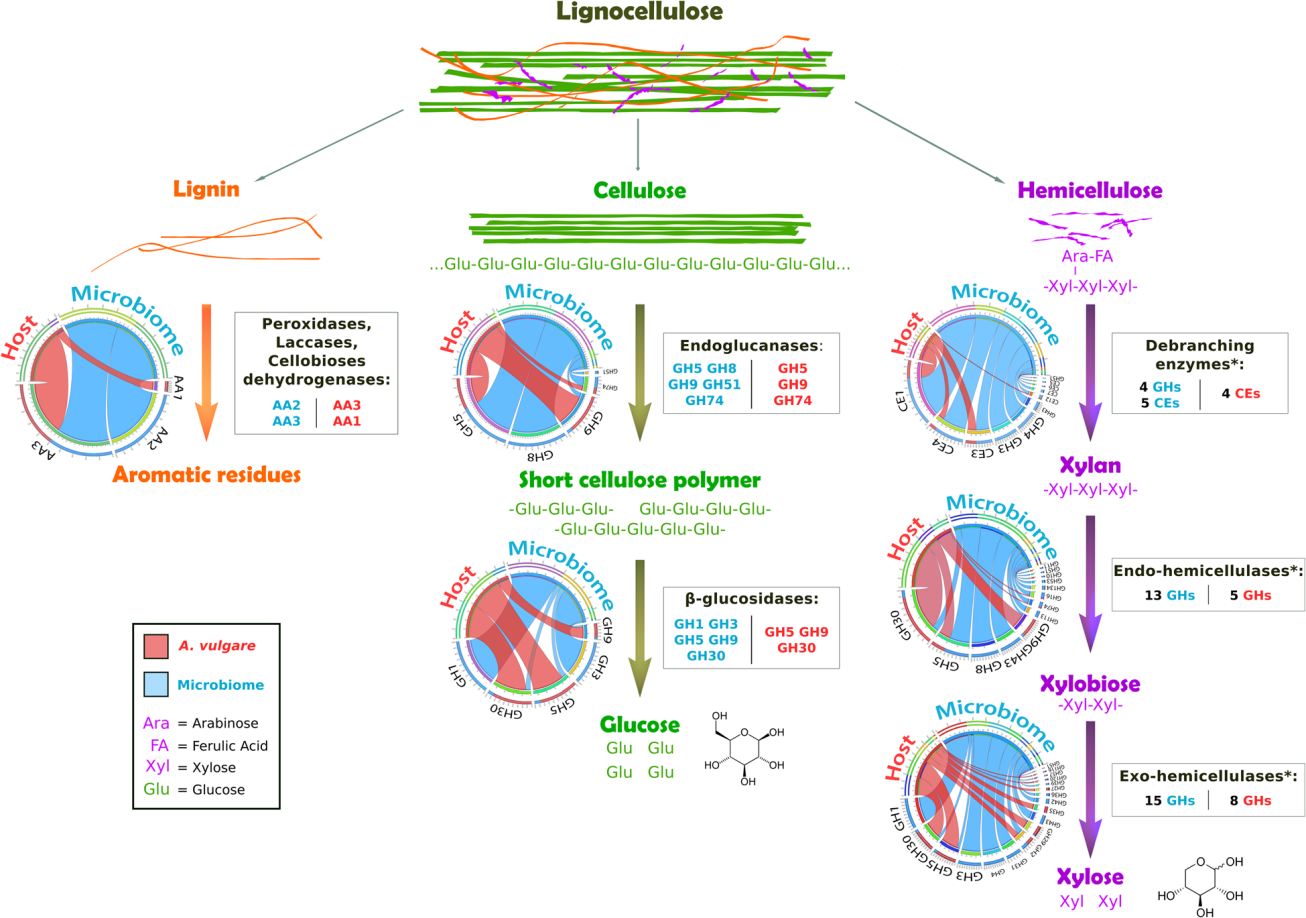
Model for lignocellulose degradation in the *A. vulgare* holobiont. Diagrams represent the CAZy families contributed by the host (red) and the microbiome (blue). (I) Lignin would be partially degraded to release cellulose and hemicellulose. (II) Cellulose would be degraded by the action of endoglucanases and β-glucosidases. A high number of β-glucosidases and mechanical fragmentation by *A. vulgare* could compensate for the lack of exoglucanases. (III) The *A. vulgare* holobiont could degrade most types of hemicellulose due to the high diversity of Debranching enzymes* (CE1, CE3, CE4, CE5, CE6, CE7, CE12, GH3, GH4, GH43, GH51), Endo-hemicellulases* (GH5, GH8, GH9, GH10, GH11, GH16, GH30, GH43, GH51, GH53, GH74, GH113, GH128, GH134), and Exo-hemicellulases* (GH1, GH2, GH3, GH4, GH5, GH27, GH29, GH30, GH31, GH35, GH36, GH39, GH42, GH43, GH51, GH57, GH116, GH120)

**Fig. 2:**
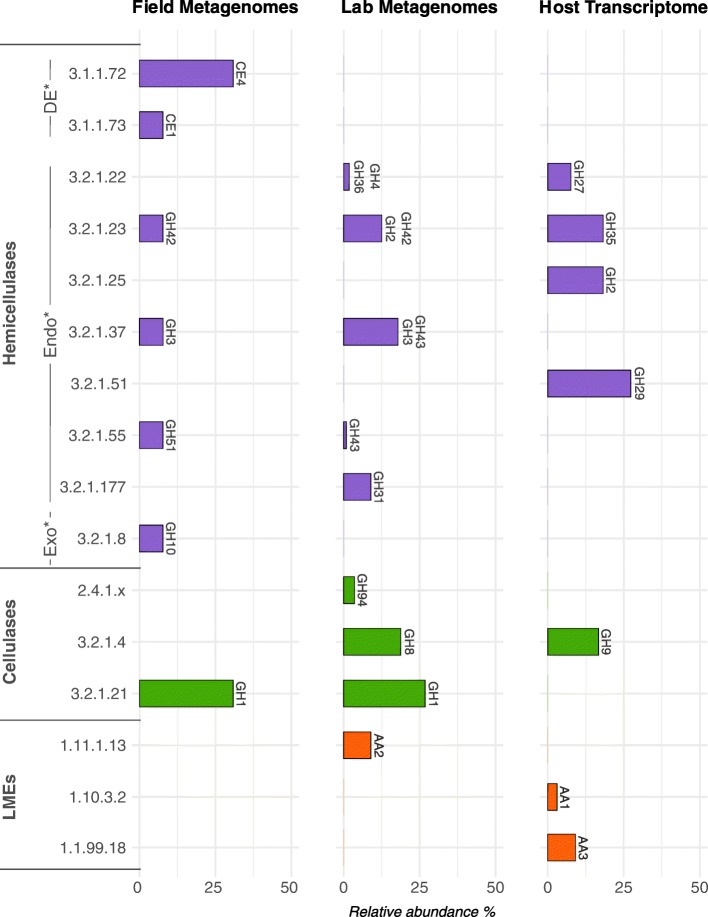
Prediction of enzymatic functions (EC number) of debranching enzymes (DE), endo-hemicellulases (Endo), exo-hemicellulases (Exo), cellulases, and lignin modifying enzymes (LMEs) identified in the metagenomes of specimens from the field and the laboratory and in the host transcriptome. Relative abundance (in %) for a given predicted enzymatic function was calculated by dividing the identified counts for a given enzyme by the total counts identified in a metagenome or in the transcriptome

Three AA families known as lignin modifying enzymes (LMEs) were found in the *A. vulgare* holobiont (Table 3, Fig. 1): laccases (AA1; EC 1.10.3.2) and cellobiose dehydrogenases (AA3; EC 1.1.99.18) were identified in the host, and peroxidases (AA2; EC 1.11.1.13) were identified in the microbiome (Fig. 2).

Cellulases are commonly classified as GH families. Nine GH families known to exhibit cellulase activity were identified in the *A. vulgare* holobiont (Table 3, Fig. 1). They were all present in the microbiome and four were also found in the host (GH5, GH9, GH30, GH74). Among them, two GH families (GH1 and GH3) were identified as glucosidases (EC 3.2.1.21) in the microbiome by Hotpep (Fig. 2), and another two were identified as endoglucanases (EC 3.2.1.4) in the microbiome and in the host (GH8 and GH9, respectively). Furthermore, genes encoding cellobiose phosphorylase (GH94; EC 2.4.1.20) were found in the microbiome.

Hemicellulases were highly represented in the *A. vulgare* holobiont, corresponding to many CE and GH families. Thirty-two were identified in the microbiome and 15 in the host (Table 3, Fig. 1). All hemicellulases found in the host transcriptome except for GH116, GH27, and GH35 were also present in the microbiome. Among them, Hotpep identified two debranching enzymes, seven exo-hemicellulases, and one endo-hemicellulase (Fig. 2).

The *A. vulgare* holobiont presented a high diversity of lignocellulose-binding modules (Table 3). The majority (93.9%) was present in the microbiome; 39 CBMs found in the microbiome are known to bind diverse components present in lignocellulose, while only 8 lignocellulose-binding module families were found in the host. Furthermore, all CBM families found in the host were also present in the microbiome.

**Fig. 3:**
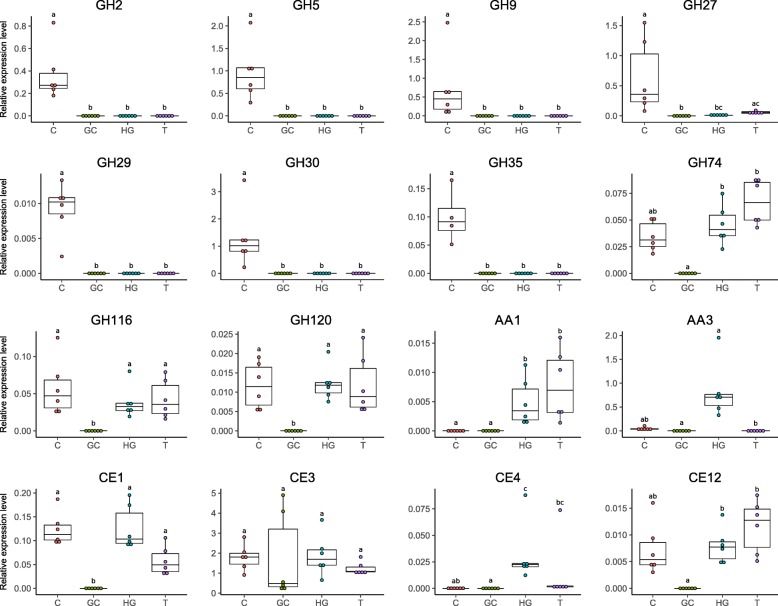
Quantitative RT-PCR analysis of the expression of representative host lignocellulose-degrading CAZymes in caeca (C), gut content (GC), hindgut (HG), and non-digestive tissues (T). Transcripts with the highest RPKM value were chosen to represent each family of interest. Expression of each gene was normalized based on the expression of Ribosomal Protein L8 (RbL8) and Elongation Factor 2 (EF2) as reference genes. Different letters indicate statistically significant differences (*p* < 0.05) after Kruskal–Wallis rank sum test

The expression of lignocellulose-degrading CAZymes identified in the host transcriptome was confirmed by RT-qPCR (Fig. 3). Specifically, one representative gene with the highest RPKM value was selected for each CAZy family identified in the host transcriptome and its expression quantified in digestive tissues (caeca, hindgut, gut content) and non-digestive tissues. All selected genes were expressed in vivo except for GH16, which we were not able to amplify. Most genes encoding glycoside hydrolases (excepting GH74, GH116, GH120), including cellulases and hemicellulases, were specifically expressed in the caeca, whereas genes encoding AA3 (LME) and CE4 (debranching enzyme) were highly expressed specifically in the hindgut. Other selected genes (GH74, GH116, GH120, CE1, and CE12) were ubiquitously expressed in host tissues but not in the gut content. Finally, CE3 was expressed in all host tissues as well as the gut content. Given that GH74, GH116, and GH120 host transcripts were expressed in all tissues and that they were not identified as lignocellulose-degrading CAZymes by Hotpep (Additional file 6: Table S4), they were excluded from the rest of the study.

### Microbiota associated with lignocellulose degradation

In order to assign genes annotated as lignocellulose-degrading CAZymes and lignocellulose-binding modules in the microbiome to prokaryotic taxa, these genes were compared against the non-redundant protein database by BLASTp searches and the results were imported into MEGAN6 [70]. This allowed the taxonomic identification of 438 genes, corresponding to 95.4% of all prokaryotic genes encoding lignocellulose-degrading CAZymes in the metagenome assemblies. Concerning the lignocellulose-binding modules, only 8.9% (78 genes) of these modules were taxonomically assigned to prokaryotic genes. Most lignocellulose-degrading genes and lignocellulose-binding module genes were associated with the bacterial phyla Proteobacteria and Actinobacteria (Table 5). The remaining bacterial genes were distributed among the Bacteroidetes, Firmicutes, and several candidate phyla. Several lignocellulose-degrading genes were also assigned to archaea belonging to the phylum Thaumarchaeota (Table 5).

**Table 5:** Prokaryotic phyla associated with genes contributing to lignocellulose degradation in the isopod metagenomes depending on host origin. Presented are the total numbers of CAZymes in metagenome assemblies

Phylum	Lignocellulose-degrading genes		Lignocellulose-binding module genes	
	Field	Lab	Field	Lab
Proteobacteria	38	321	14	24
Bacteroidetes	2	1	2	–
Actinobacteria	30	–	16	3
Firmicutes	1	–	–	–
Bacteria candidate phyla	3	–	3	–
Thaumarchaeota	29	–	2	–
Unclassified bacteria	3	10	3	11

**Fig. 4:**
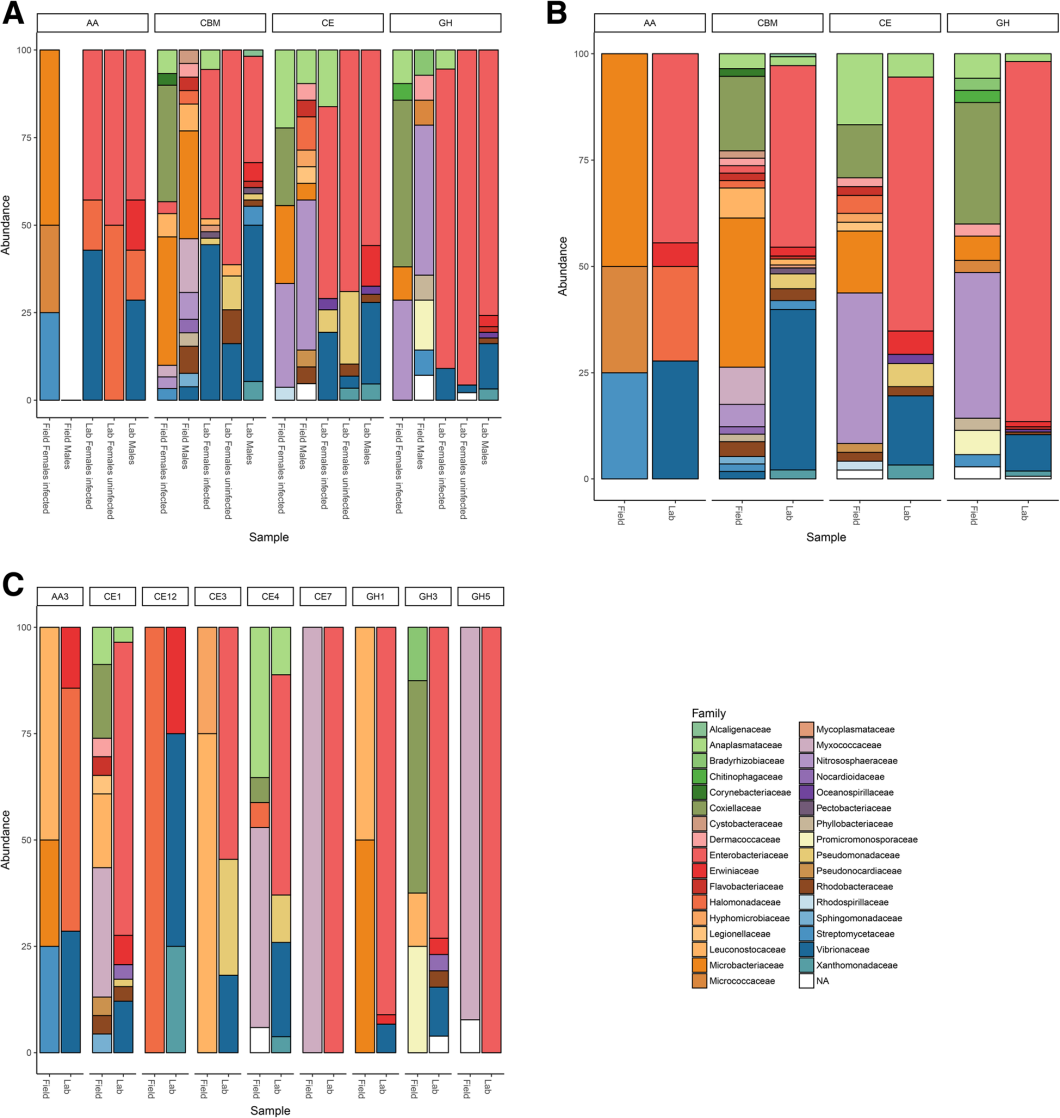
Relative abundance of prokaryotic taxa contributing lignocellulose-degrading CAZymes depending on **a** host origin, gender, and *Wolbachia* infection status, **b** host origin alone, and **c** for several genes consistently present in isopods of both field and laboratory origin. See Table 3 for a detailed annotation of these genes

The lignocellulose-degrading microbiotas from laboratory lineages (consisting of *Wolbachia-*infected females, uninfected females and males) were highly similar (Fig. 4a). In contrast, the microbiotas of males and females from the field were different, and this was due to the high abundance of *Rickettsiella* (Coxiellaceae family) in females, while the other bacterial families were similarly abundant in both sexes (Fig. 4a). Therefore, samples from both sexes and with different *Wolbachia* infection status in the two populations were grouped for further analyses (Fig. 4b).

**Fig. 5:**
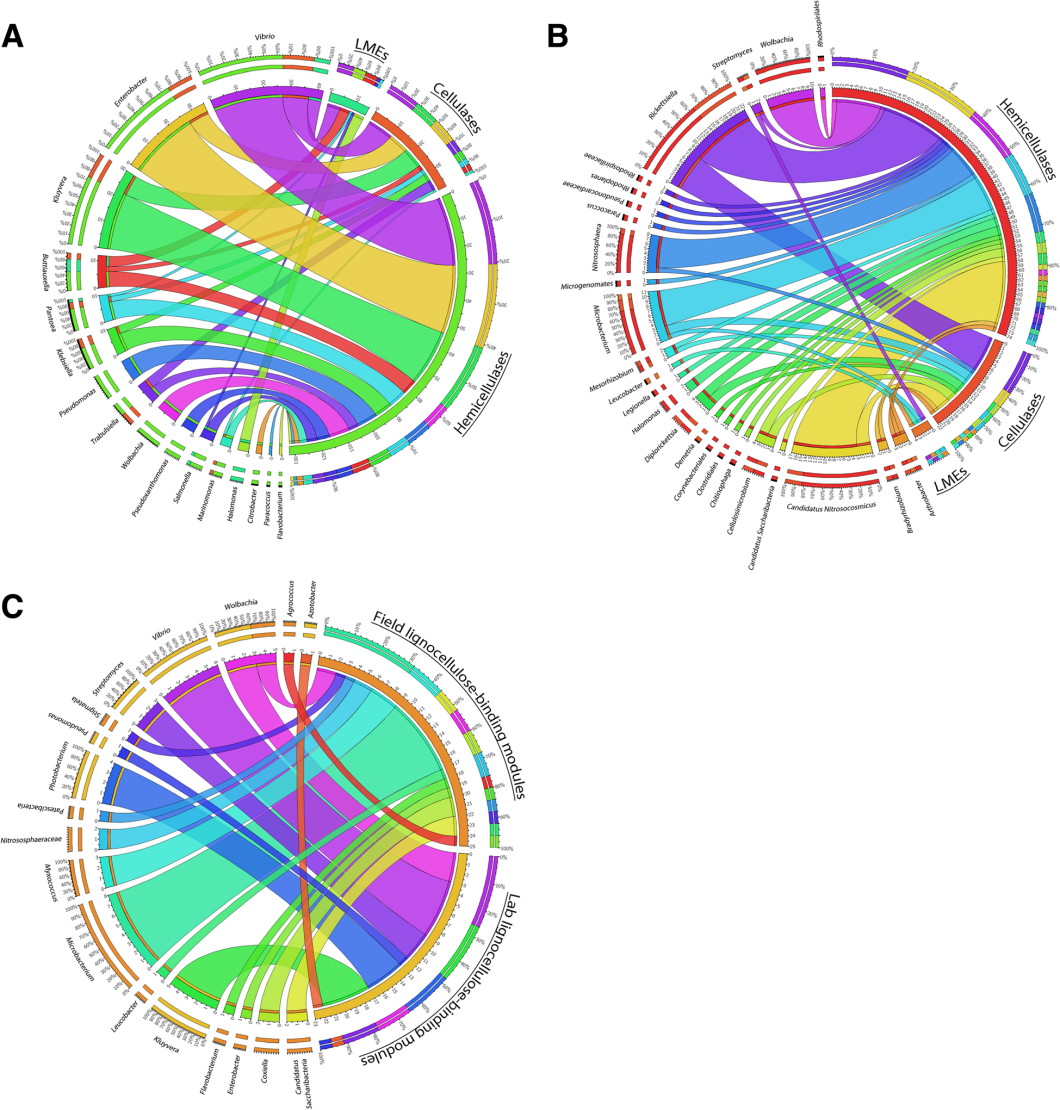
Lignocellulose-degrading enzymes and their associated microbial community in (**a**) isopods from the laboratory and (**b**) isopods from a natural population. **c** The microbial taxa contributing lignocellulose-binding modules in both field and laboratory specimens

There was a clear difference in the contribution of different microbial taxa to lignocellulose degradation between field and lab-derived isopods (Figs. 4 and 5). Enterobacteriaceae and Vibrionaceae were the bacterial families with the highest contribution of lignocellulose-degrading genes in animals from laboratory lineages (74.1% of laboratory lignocellulose-degrading genes), whereas Coxiellaceae, Nitrososphaeraceae, Microbacteriaceae, and Anaplasmataceae contributed more lignocellulose-degrading genes in isopods from the field population (49.7% of field lignocellulose-degrading genes). In particular, the genera *Vibrio*, *Kluyvera*, and *Enterobacter* contributed most hemicellulases and cellulases in specimens from the laboratory, whereas *Vibrio*, *Buttiauxella*, and *Halomonas* contributed most LMEs (87% of the bacterial genes encoding LMEs) (Fig. 5a). In isopods from the field, the bacteria *Rickettsiella*, *Wolbachia*, *Microbacterium*, and the archaea *Candidatus* Nitrosocosmicus and *Nitrososphaera* contributed 72% of the microbial genes encoding hemicellulases (Fig. 5b). Similarly, the bacteria *Rickettsiella*, *Microbacterium*, *Cellulosimicrobium*, and the archaea *Candidatus* Nitrosocosmicus were the microorganisms most frequently associated with cellulases (77% of prokaryotic genes encoding cellulases in isopods from the field) (Fig. 5b). Finally, *Streptomyces*, *Microbacterium*, *Arthrobacter*, and *Leucobacter* were associated with all genes encoding LMEs in isopods from the field.

Among the 78 genes encoding lignocellulose-binding modules that were assigned to prokaryotes, the bacterial genera *Microbacterium* and *Myxococcus* contributed most genes in isopods from the field (44%), while *Kluyvera*, *Vibrio*, and *Photobacterium* represented 65% of prokaryotes associated with lignocellulose-binding modules in laboratory samples (Fig. 5c).

Given that fungi play an important role in lignocellulose degradation in various ecosystems, their presence in the metagenomes of *A. vulgare* was also expected. Eleven fungal genes encoding lignocellulose-degrading CAZymes were detected in only one metagenome (the gut content of *Wolbachia*-free females from the laboratory lineages), and they were all affiliated with *Aureobasidium pullulans*, a ubiquitous fungus that can be found in various ecosystems and in association with plants [77]. The low number of lignocellulose-degrading CAZymes identified from fungi may be due to the prokaryotic enrichment procedure used for the construction of the metagenomic libraries.

Although some CAZymes were specific to a particular sample type (Table 3), overall there was a high functional redundancy in enzymatic activities between microbiotas from lab- and field-derived isopods, indicating that several CAZy families could have the same enzymatic activity. Furthermore, all shared CAZy families were associated with different microbiotas depending on host origin (Fig. 4c). Thus, different microbial communities would still provide the same enzymatic activities for lignocellulose degradation.

## Discussion

This study represents the first investigation of the complete enzyme repertoire involved in lignocellulose degradation in a terrestrial isopod. The construction of a highly complete host transcriptome combined with a functional characterization of the microbiome of *A. vulgare* via shotgun metagenomics allowed the identification of both endogenous and microbial enzymes degrading lignocellulose. *A. vulgare* of both laboratory and field origin as well as several host tissues and the gut content were included, with the aim to assess potential impacts of the environment and diet. We produced a highly complete reference transcriptome of *A. vulgare* (95.7% of complete genes identified from BUSCO database) and 15 metagenome assemblies allowing a deep investigation of the *A. vulgare* microbiome. Many CAZymes belonging to 231 families were detected in the *A. vulgare* holobiont, placing this species at the same level as termites in terms of CAZyme diversity [78, 79]. Among these, we identified a high diversity of lignocellulose-degrading enzymes, including cellulases, hemicellulases, and LMEs.

The synergistic action of several specific CAZymes is needed to completely degrade lignocellulose. Among CAZy classes, several families are known to be particularly implicated in lignocellulose degradation: glycoside hydrolase families (GHs), carbohydrate esterase families (CEs), and auxiliary activity families (AAs) [65]. Prior to cellulose and hemicellulose decomposition, the complex heteropolymer lignin has to be degraded. Lignin is the most difficult component to degrade in lignocellulose because of its complex and irregular structure, which requires enzymes (LMEs classified in AA families [12, 65]) with less specificity than cellulases and hemicellulases [80]. To date, the lignin degradation mechanism in *A. vulgare* is not well understood: although Zimmer and Brune [81] showed that the isopod gut presents adequate conditions for aerobic and oxidative digestive processes that are needed for lignin degradation, lignin seems to be only partially degraded in isopods [3, 30, 34, 82, 83]. While LMEs are uncommon in animals, we identified laccases (AA1) and cellobiose dehydrogenases (AA3) in the *A. vulgare* transcriptome. Our results further demonstrated that endogenous LMEs are expressed in the *A. vulgare* hindgut, which agrees with termites where endogenous laccases are expressed throughout the whole gut [23]. The absence of LME expression in the caeca is probably due to the too low oxygen level in this tissue [46]. Laccases seem to be expressed exclusively by *A. vulgare* itself, while the microbiota provides additional cellobiose dehydrogenases (AA3) and peroxidases (AA2). Although the role of endogenous laccases in lignin degradation is well characterized in termites [23, 24, 27], we show here that a laccase gene (belonging to the AA1 family) was also expressed in *A. vulgare* non-digestive tissues, suggesting a role beyond lignin degradation. Indeed, laccases can also be involved in both the melanization and sclerotization of the cuticular layers, as shown in other arthropods [84]. Cellobiose dehydrogenases were found in both the host and the microbiome, but their exact role in lignocellulose degradation is still unclear, with some evidence for their implication in both cellulose and lignin degradation [85, 86]. Our results provide new insights into lignin degradation in terrestrial isopods, demonstrating that a cooperation between members of the *A. vulgare* holobiont could indeed result in the partial modification of lignin and the release of cellulose and hemicellulose. As previously demonstrated in termites [23, 87, 88], this could be achieved in a cooperative manner in different parts of the digestive system.

Once lignin is degraded, enzymes can attack cellulose and hemicellulose from plant biomass. Cellulose is the most abundant component of lignocellulose [19]. Its degradation is best characterized in fungi where three types of enzyme are required for the process: endoglucanases, β-glucosidases, and cellobiohydrolases (exoglucanases) [89]. Our comparative analysis of transcriptomic and metagenomic datasets suggests that *A. vulgare* could digest cellulose in cooperation with its microbiota, similar to termites [23–25, 27]. Previous studies had already characterized endogenous endoglucanases in isopods, but not in their microbiome [47–49]*.* Endoglucanases annotated in the *A. vulgare* reference transcriptome were affiliated with the GH5 and GH9 families. The GH9 family comprises the most widespread endogenous animal endoglucanases, whereas GH5 is less common [90]. Endoglucanases were also found in great abundance in the microbiome of *A. vulgare*, belonging to the GH5, GH8, GH9, GH51, and GH74 families. Whereas endoglucanases are widespread in animals, β-glucosidase are less common [90]. Among the CAZy families known as β-glucosidases identified in the *A. vulgare* reference transcriptome (GH5, GH9, and GH30) and in the microbiome (GH1 and GH3), only GH1 genes were functionally predicted as β-glucosidases in the microbiome. The third type of cellulolytic enzymes, cellobiohydrolases are uncommon in animals and are usually classified as GH6, GH7, and GH48 [19, 65, 78, 91]. Accordingly, there was no clear evidence for the presence of cellobiohydrolases in the *A. vulgare* holobiont. However, Allardyce et al. [92] proposed a model for cellulose hydrolysis involving only endoglucanases and β-glucosidases in the land crab *Gecarcoidea natalis*. As in several insects, the lack of cellobiohydrolases could thus be compensated by the high number of endoglucanases, despite the low activity of these enzymes against crystalline cellulose [93]. Furthermore, mechanical fragmentation of the food into small particles by mandibles and proventriculus [94] facilitates enzyme access to lignocellulose [19]. The identification of host endoglucanases in the caeca of *Porcellio scaber*, the common rough woodlouse [48], shed doubts on the previous hypothesis [95] that cellulose degradation was achieved by endosymbiotic bacteria in the caeca. Zimmer and Topp [95] showed that cellulase activity was high in caeca and hindgut, but they could not clearly attribute this activity to the microbiota, the isopod itself, or both. Our gene expression analysis reveals that the isopod caeca represent the major site of transcription of endogenous host cellulases, while very few microbial cellulases were found in caeca. Indeed, the majority of microbial cellulases were found in the gut content. These results suggest a two-step collaboration for cellulose digestion, where the pill bug primarily hydrolyses cellulose with its own endoglucanases produced in the caeca and the gut microbiota completes cellulose digestion with other endoglucanases and β-glucosidases in the hindgut as in termites [21, 23].

Hemicellulases have been previously characterized in several crustaceans [96–98], but not yet in terrestrial isopods. The main chain of hemicellulose is composed of xylose, glucose, and mannose, which is often branched with arabinose, galactose, and other acidic sugars. Therefore, the degradation of hemicellulose requires a larger enzymatic arsenal than the degradation of cellulose. Our metagenome and transcriptome data revealed that the gut microbiota and the host produce many hemicellulases, again suggesting a close cooperation between members of the *A. vulgare* holobiont in hemicellulose degradation. We identified 31 hemicellulase families in the *A. vulgare* holobiont, which could degrade most types of hemicellulose. Based on our data, the microbiota would play the major role in hemicellulose degradation, providing more than twice as many hemicellulose families than the host. The comparative analysis of the hemicellulases from the host and the microbiome further revealed a high level of functional redundancy with multiple predicted xylanases, arabinases, mannanases, and xyloglucanases. As observed in other studies [4], this redundancy may indicate an enzymatic synergism between *A. vulgare* and its microbiome, which might degrade hemicellulose in a cooperative manner.

In addition, we identified the microbial taxa contributing genes potentially involved in lignocellulose degradation in *A. vulgare*. Previous work had already demonstrated a high bacterial diversity in all major tissues of *A. vulgare*, with distinct bacterial communities between individuals originating from the field or from the laboratory [42, 51, 52]. Here, we show that despite their different taxonomic compositions, these bacterial communities are similar in their repertoire of lignocellulose-degrading CAZymes, resulting in a high functional redundancy for lignocellulose degradation between field and laboratory-derived isopods. Indeed, many CAZy families can act on multiple substrates and many enzymatic activities can be provided by several CAZy families [65, 99]. While bacteria such as Fibrobacteres, Bacteroidetes, and Firmicutes abundantly colonize the hindgut of termites [28, 100], the composition of the bacterial communities associated with lignocellulose-degradation in *A. vulgare* was significantly different. Being dominated by Proteobacteria and Actinobacteria, the lignocellulose-degrading microbiota of *A. vulgare* was in fact more similar to the gut microbiota of xylophagous beetles [101]. Proteobacteria were particularly dominant in *A. vulgare* from laboratory lineages. Among them, Enterobacteriaceae (genera *Klebsiella*, *Enterobacter*, and *Buttiauxella*) contributed most lignocellulose-degrading CAZymes. These bacteria are common in arthropods [102] and known to have various metabolic capabilities, such as contributing to nitrogen intake and lignocellulose degradation in insects [101]. The second largest contribution of lignocellulose-degrading CAZymes in *A. vulgare* from laboratory lineages was provided by members of the Vibrionaceae, also Proteobacteria*.* These bacteria are best known for their pathogenicity, and to date, there is only one study reporting cellulases in the *Vibrio* genus [103]. *Halomonas* spp. (Halomonadaceae) are also of interest, since they represent one of the most abundant bacterial genera within the *A. vulgare* microbiome [42, 51, 52].Their genomes possess several genes encoding LMEs, and they are known to contribute to lignin degradation [104]. Accordingly, they were identified as major contributors of LMEs in isopods from laboratory lineages. Among the bacteria associated with genes encoding lignocellulose-degrading CAZymes in isopods from the field, several Actinobacteria such as *Microbacterium* spp. (Microbacteriaceae) and *Cellulosimicrobium* spp. (Promicromonosporaceae) have previously been shown to possess cellulose and hemicellulose-degrading activities [105–107]. Similarly, bacteria associated with genes encoding LMEs in isopods from the field were already known for their lignin-degrading activity: *Arthrobacter* spp. (Micrococcaceae), *Streptomyces* spp. (Streptomycetaceae), *Microbacterium* spp., and *Leucobacter* spp. (Microbacteriaceae) [108, 109]. Our results further showed that Archaea might also contribute to lignocellulose degradation in *A. vulgare*: we identified genes encoding cellulases and hemicellulases from *Candidatus* Nitrosocosmicus and *Nitrososphaera*, two genera of the Nitrososphaeraceae family. To date, no role in lignocellulose-degradation has been demonstrated for these archaea, but it has been suggested that they might contribute to nitrification in fertilized soils and oxidize ammonia [110, 111].

Several lignocellulose-degrading CAZymes were associated with two unexpected bacteria, *Rickettsiella* and *Wolbachia*. Indeed, many hemicellulases and cellulases were identified as belonging to the genus *Rickettsiella* (Coxiellaceae) in *A. vulgare* from the field. *Rickettsiella* spp. are mainly known as arthropod pathogens (reviewed in [112]) or mutualists [113], and until now, no lignocellulose-degrading activity has been demonstrated for these bacteria. In addition, several acetyl xylan esterases (endo-hemicellulases) belonging to the CE4 family were associated with *Wolbachia* spp. (Rickettsiaceae) in isopods harboring feminizing *Wolbachia* strains, independent of host origin. The CE4 family comprises CAZymes which have many other catalytic activities, such as chitin deacetylase, chitooligosaccharide deacetylase, and peptidoglycan deacetylase [65]. Therefore, CE4 enzymes do not necessarily contribute to hemicellulose degradation. The only case where *Wolbachia* is known to play an essential nutritional role for its host is the obligate symbiosis between *Wolbachia* and the bed bug (*Cimex lectularius*), with *Wolbachia* providing vitamin B [114–117]. Its role in lignocellulose degradation therefore remains to be experimentally tested. In contrast, no lignocellulose-degrading CAZymes associated with *Candidatus* Hepatoplasma spp. were identified in this study. These bacteria are widespread facultative symbionts residing in the caeca of terrestrial isopods, and they had been initially thought to be involved in lignocellulose-degradation [41, 42, 46]. However, the genome of *Candidatus* Hepatoplasma from *A. vulgare* does not contain any lignocellulose-degrading CAZymes [45]. Nonetheless, the fact that *Candidatus* Hepatoplasma increases its host’s survival on a cellulosic low-quality diet [118] still suggests a nutritional role of the symbiont, although it may not be linked to lignocellulose degradation.

## Conclusion

In accordance with the hypothesis of Zimmer et al. [38, 39], our study provides new insights into the contribution of the microbiota to the digestion of terrestrial food sources, which may have enabled the colonization of land by terrestrial isopods. We demonstrate that there is a potential collaboration between *A. vulgare* and its microbiome for an efficient lignocellulose digestion. Despite distinct bacterial communities depending on host origin, microbial functions related to lignocellulose degradation are highly similar between laboratory lineages and natural isopod populations. These functionally redundant bacterial communities may thus have evolved with the shift in the host’s diet [119, 120], along with digestive mechanisms of the host. However, more detailed functional investigations based on experimental approaches as well as metatranscriptomics or metaproteomics will be necessary to validate the specific functional contributions of each member of the woodlice holobiont. Moreover, the extension of this work to other isopod species will further improve our understanding whether shifts in the host-associated microbiota might indeed have influenced the successful colonization of land by terrestrial isopods.

## Additional files

Additional file 1: **Table S1.** List of primers used in RT-qPCR*.* (XLSX 10 kb)

Additional file 2: **Table S2.** List of CAZymes identified in metagenome assemblies and in the transcriptome of *A. vulgare.* (XLSX 18 kb)

Additional file 3: Protein sequences of identified CAZy genes in metagenome assemblies. (FA 596 kb)

Additional file 4: Protein sequences of identified CAZy genes in *A. vulgare* transcriptome. (FA 861 kb)

Additional file 5: **Table S3.** RPKM values for CAZymes identified in the transcriptome of *A. vulgare.* (XLSX 50 kb)

Additional file 6: **Table S4.** List of predicted enzymatic functions of CAZymes identified in metagenomes and host transcriptome. (XLSX 14 kb)

Additional file 7: **Table S5.** Comparison of the glycoside hydrolase (GH) profiles of *A. vulgare*, termite, snail, slug, human, panda, HF cross, jersey cow, cow, reindeer, and macropod gut metagenomes, showing GH groups that are involved in the breakdown/modification of plant cell wall polysaccharides. (XLSX 14 kb)
